# Torque Teno Virus (TTV) Among β-Thalassemia and Haemodialysis Patients in Mazandaran Province (North of Iran)

**Published:** 2017-02-28

**Authors:** Hossein Jalali, Mohammad Reza Mahdavi, Najmeh Zaeromali

**Affiliations:** 1 *Students Research Committee, Thalassemia Research Center, Mazandaran University of Medical Sciences, Sari, Iran.*; 2 *Thalassemia Research Center, Mazandaran University of Medical Sciences, Sari, Iran.*; 3 *Department of Bacteriology and Virology, Shiraz Medical School, Shiraz University of Medical Sciences, Shiraz, Iran.*

**Keywords:** Torque Teno virus (TTV), β-thalassemia, haemodialysis

## Abstract

Torque Teno virus (TTV) is a transfusion transmitted virus that seems to be involved in several complications such as acute respiratory diseases, liver diseases, AIDS, cancer, and autoimmune reactions. In the present study the frequency of TTV was investigated among β- thalassemia (BT) and haemodialysis (HD) patients (high risk patients for TTV) in Mazandaran province, Iran. DNA was extracted from the serum of 82 BT and 100 HD patients, and nested PCR method was applied to detect TTV DNA. The aspartate transaminase(AST) and alanine transaminase (ALT) enzyme levels in BT patients were measured using photometric assay. The mean age of BT and HD patients as 23.4±5.4 and 48.8±8.2 years, respectively. 21% of HD and 26.8% of BT patients were infected with TTV, respectively. The frequency of TTV was not significantly different between two groups of patients and there was no significant correlation between sex and TTV infection. The mean AST and ALT levels in TTV positive BT patients was not significantly higher than TTV negative cases. The present study showed that TTV prevalence in BT patients with recurrent blood transfusion was not significantly higher than HD patients. The investigation of TTV prevalence in healthy individuals is recommended to identify if transfusion or dialysis is associated with higher TTV infection. Besides, although TTV infection did not change the AST and ALT enzymes in BT patients, the liver involvement may still exist in these patients.

TTV (Torque Teno Virus) is a single strand DNA virus that was detected for the first time in a Japanese man with non A-G hepatitis at 1997 (1). The genome of the virus is 3.8 Kb long containing 2.6 kb of coding regions that translate 3 or more proteins (2-4). At first, it seemed that the virus was associated with hepatitis, but numerous studies showed that it was present in more than 50% of apparently healthy subjects in some populations (5, 6). However, the pathogenic role of TTV is not well understood. TTV is suggested to be involved in several diseases such as acute respiratory diseases (7), liver diseases (5, 6), AIDS (8), cancer (9), and autoimmune reactions (10). Nonetheless, convincing evidence that support the role of TTV in the mentioned disorders have not been reported. Since TTV has a great variety of genotypes (11), some genotypes may be responsible for pathogenesis of the virus.

Beta-thalassemia (BT) is one of the most common genetic disorders and a serious health problem in Iran, especially in Mazandaran province (12-14). Recurrent blood transfusion is the main treatment for improving clinical manifestation of the disease (15). In patients with chronic transfusion regimes, liver diseases that are mostly caused by the agents transmitted by blood products and concomitant iron liver siderosis are common. Although new viral agents and TTV are identified in these patients, their association with any disease has not been reported (16).

Infections are the major causes of mortality and morbidity in haemodialysis (HD) patients. Hence, the patients with chronic renal failure (CRF) undergoing HD are at risk of TTV infection and are suffering from mentioned complications (17).

The aim of the present study was to investigate the frequency of TTV in two groups of BT and HD patients in North of Iran. 

## Materials and methods

Eighty two BT and 100 HD patients were enrolled in the study in Mazandaran province in northern Iran. The BT patients that underwent recurrent blood transfusion therapy, registered at Thalassemia Research Center at Boali hospital, Sari, Iran, with haemoglobin (Hb) levels less than 9 g/dl, were included in the study.Likewise the HD patients with CRF registered at dialysis ward of Fatemeh Zahra hospital, Sari, Iran were also included in the study. The intermediate BT patients with Hb more than 9 g/dl that were independent of blood transfusion were excluded from the study. This study was approved by Ethics Committee of Mazandaran University of Medical Sciences. Informed consent was also obtained from the patients. All patients were assured that their information would be kept confidential.

DNA was extracted from 200 µl of the serum samples using QIAamp MinElute Virus Spin Kit (Qiagen, Germany). Nested PCR method was applied for the detection of TTV DNA in which two sets of specific primers were used for the amplification of the viral DNA (Table 1) (18).

The first round of PCR was performed in a total volume of 25 μl containing 5 μl of extracted DNA, 10 pmole of each primer, 2 mM Mgcl2, 200 μM dNTPs, 2.5 μl of 10X PCR buffer and 1 unit of Taq DNA polymerase (Thermo Science, Germany). PCR program was as follows: initial denaturation at 95ºC for 5 min, 35 cycles including 94ºC for 1 min, 58 ºC for 1 min and 72 ºC for 1 min, and final extension at 72 ºC for 6 min. 2 μl of the first round PCR product was applied as a template for the second round of PCR that contained NS3 and NS4 primers, and amplification was accomplished with cycling conditions identical to the first PCR run. The PCR products were analyzed using electrophoresis on 1% agarose gel and were visualized under UV illumination.

The serum levels of aspartate transaminase (AST) and alanine transaminase (ALT) enzymes were measured in patients with BT by an automated photometric assay method using a commercial kit (Pars Azmoon, Iran) on Hitachi 917 system (Japan).

**Table 1:** The sequence of primers used for TTV DNA detection

	**Product size (bp)**	**Primer name**	**Primer sequences 5`→3`**
First round of PCR	320	NS1	GGGTGCCGAAGGTGAGTTTAC
		NS2	GCGGGGCACGAAGCACAGAAG
Second round of PCR	295	NS3	AGTTTACACACCGAAGTCAAG
		NS4	AGCACAGAAGCAAGATGATTA

Independent sample t–test and chi-square tests applying SPSS software version 16 (U.S.A) were used for statistical analysis.

## Results

Eighty–two BT patients including 38 (46%) male and 44 (54%) female cases and 100 HD patients including 41 (41%) female and 59 (59%) male subjects were studied. The mean age of BT patients was 23.4±5.4 years (range 5-35) and the mean Hb levels of the patients was 7.9± 2 g/dl. The mean age of the HD patients was 48.8± 8.2 years (range 25-68). TTV was detected on 22 out of the 82 (26.8%) BT and 21 out of 100 (21%) HD patients (Table 1). The results of chi-square test showed that the frequency of TTV infection between BT and HD patients was not significantly different (P=0.38). There was no significant relationship between gender and TTV infection (P=0.12).

**Table 2:** Frequency of TTV infection between BT patients with multiple transfusion and HD patients

	**Age** **Mean±SD**	**TTV Positive (%)**				**TTV Negative (%)**			
		**Male** **n(%)**	**Female** **n(%)**	**AST** **Mean±SD**	**ALT** **Mean±SD**	**Male** **n(%)**	**Female** **n(%)**	**AST** **Mean±SD**	**ALT** **Mean±SD**
BT patients (n=82)	23.4±5.4	10 (45%)	12 (55%)	22.00±12	20.7±16	28 (46.5%)	32 (53.5%)	29.6±17	31.6±25
HD patients (n=100)	48.8±8.2	9 43%)	12 (57%)	NI	NI	32 (40.5%)	47 (59.5%)	NI	NI

AST: aspartate transaminase; ALT: alanine transaminase; SD: standard deviation; NI: not investigated; TTV= Torque Teno virus.

**Fig. 1:**
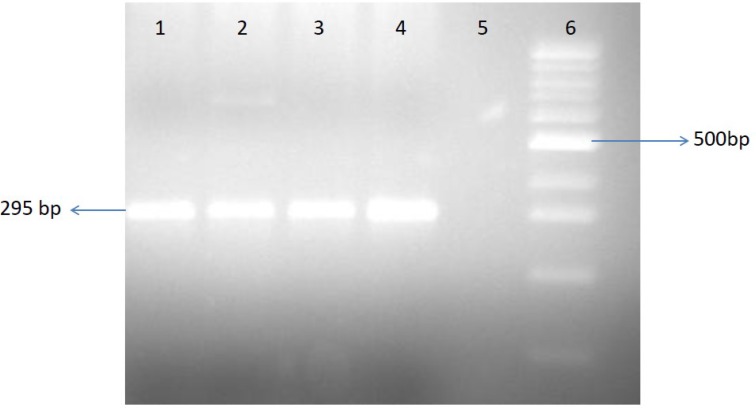
TTV detection by electrophoresis on agarose gel. Lanes 1, 2, 3, and 4: patients with TTV infection; lane 5: a patient without TTV infection; lane 6: 100 bp ladder

The mean levels of AST and ALT enzymes in BT patients were 27.6± 16 and 28.8± 23 U/L, respectively. The serum levels of these enzymes in TTV positive cases were not higher than TTV negative patients (P=0.21) (Table 1).

## Discussion

The prevalence of TTV is varied among BT patients from different populations. In the current study, the frequency of TTV in BT patients with recurrent blood transfusion and in HD patients were investigated.

The investigation of TTV in Khuzestan province (Southwest of Iran) showed that 57.2% of BT patients (143 out of 250) and 20% of healthy controls (54 out of 250) were positive for TTV and its prevalence was significantly different between these two groups (P<0.0001). The frequency of TTV infection in Mazandaran was much lower than Khuzestan province among BT patients (26.8% vs. 57.2%) (19). Another study in south of Iran indicated that of the 452 BT patients, 160 (35.4%) were positive for TTV and mean ALT and AST values in TTV positive patients were significantly higher than TTV negative ones (20). Nevertheless, in the present study these values were not significantly different. Analysis of 324 Iranian Azeri Turkish patients on maintenance HD in 2005 indicated that 9.3 % of the patients were infected with TTV and similar to the present study, there was no association between TTV and ALT levels (21). In Isfahan province, the prevalence of TTV in injection drug users (58%) was significantly higher than HD patients (17 %) and healthy individuals (8%) (22).

Several studies indicated that TTV was highly present in BT patients in Turkey (23) (61%) and Italy (73% in the BT children and 69.1% in adult BT) (24, 25). These results suggest that TTV-DNA is transmitted to the recipients by blood and blood products. So, blood transfusion is one of the most ways for TTV transmission.

In Saudi Arabia, the prevalence of TTV in HD patients was high and statistically significant; 42.9% compared with 19% in the control group. While similar to our results, AST and ALT levels were not significant predictors of TT virus in HD patients (26). In Italy, the prevalence of TTV DNA in dialysis patients [35/85 (41.7%)] was significantly higher than healthy population [7/65 (10.7%)] (27). Although, in the present study, the frequency of TTV in HD patients was almost half of that reported in Italy and Saudi Arabia.

The results of the present study showed that in comparison with HD patients, the recurrent blood transfusion did not increase TTV prevalence in BT patients. Yet, the investigation of TTV prevalence in healthy individuals is required to identify whether transfusion or dialysis are associated with higher TTV prevalence. Besides, even though TTV infection did not change AST and ALT enzymes in BT patients, further studies analyzing the potential role of TTV in post-transfusion hepatitis are recommended.

## Conflict of Interest

The authors declared no conflict of interest.
